# Enhanced brain image security using a hybrid of lifting wavelet transform and support vector machine

**DOI:** 10.1038/s41598-025-92580-x

**Published:** 2025-03-20

**Authors:** Asmaa Fathallah Mohamed, Ahmed S. Samra, Bedir Yousif, Abeer Tawkol Khalil

**Affiliations:** 1 Electronics and Communications Department, Faculty of Engineering, Mansoura University, Mansoura, 35516 Egypt; 2 Electrical Engineering Department, Faculty of Engineering, Kafrelsheikh University, Kafrelsheikh, 33516 Egypt; 3 Electrical Engineering Department, Faculty of Engineering and Information Technology, Onaizah Colleges, 51911 Onaizah, Al Qassim Saudi Arabia

**Keywords:** ROI and NROI, SVM, Brain image, Watermarking, Lifting wavelet transform, And geometric attacks, Engineering, Electrical and electronic engineering

## Abstract

Thanks to technological improvements, digital picture watermarking has emerged as a useful method for preventing unlawful use and manipulation of digital photographs. Providing robustness against geometrical assault while maintaining an adequate level of security and imperceptibility is a basic challenge in digital picture watermarking. With the use of support vector machine (SVM) and lifting wavelet transform (LWT), this study offers an effective authentication approach for digital image watermarking on medical images. To distinguish between the region of interest (ROI) and the non-region of interest (NROI) in the medical image, SVM is first employed in this article. After that, LWT is used to incorporate watermark data into the medical image’s NROI section (cover image). Additionally, a shared secret key has been used to increase the suggested scheme’s resilience. A vast image database is used to test the method’s performance in various scenarios. To determine whether the current plan was acceptable, the study examined several experimental investigations. The experimental results give a PSNR value of 67.81 dB and a structural similarity index measure value of 0.9999, Where the PSNR improvement percentage is 13.9462 dB, showing durability and imperceptibility for the proposed watermarking model.

## Introduction

The COVID-19 epidemic has highlighted the importance of multimedia technology and the internet. Attitudes towards internet-based communication technology have shifted. Private information, such as credit card information, bank account details, and health information, must be exchanged online, along with family photos in our daily lives. These days, revealing this crucial personal or business information online carries a large risk. Because it is sent across an unreliable digital medium, it is easily accessible and susceptible to manipulation, unauthorized use, and copyright infringement. In the realm of multimedia security, protection from illegal access requires verification, data consistency, and discretion. Thus, the user requires a reliable app or platform. It makes it simple and likely for someone to give their personal information. In this case, a cooperative machine learning and watermarking attempt would be a useful remedy. To improve robustness, security, data integrity, and authenticity. A reversible watermarking technique scheme (RWS) was developed in this study using lifting wavelet transform (LWT) and SVM^1^.

Since brain images store patient data for diagnosis, they are more common than any other type of image. Since a complete diagnosis depends on these images, greater security and confidentiality are required. In applications of telemedicine, sending a brain image across an open channel necessitates robust copyright and security measures. Therefore, sensitive information must always be encoded into those images with care. To achieve this, the brain image has been divided into two distinct areas: (1) The region of interest (ROI) is a significant portion of the diagnostic healthcare image; (2) (NROI) the non-region of interest is the remaining, less crucial portion of the image. A little misclassification could make it very difficult to get the patient to provide important information^2^. SVM is a novel class of machine learning (ML) techniques that can be applied as an image classifier in statistical learning theory. A small number of scholars have used SVM to watermark techniques for brain images in the transformation domain.

Three steps can be taken to enhance watermarking in brain images. The first phase is known as ROI and NROI categorization. Embedding a watermark into the host image is the second step. The extraction of watermark data is the final step. As an algorithm for machine learning in classification issues, SVM can be quite useful at the classification stage. Conversely, transform domain techniques like lifting wavelet transform (LWT), discrete cosine transform (DCT), discrete wavelet transform (DWT), integer wavelet transform (IWT), and singular value decomposition (SVD) are resistant to a variety of attacks during the embedding and extraction phases^3^. Nonetheless, the unique capacity of the lifting wavelet transforms to turn pixel data into integers sets it apart from the other methods and may prove useful for reversible models.

**Fig. 1:**
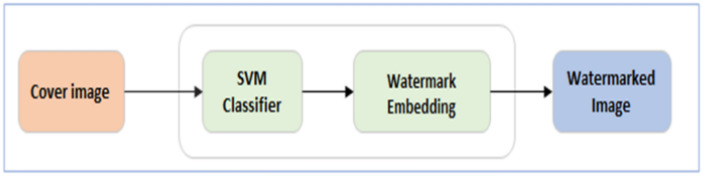
Dual-phase watermarking diagram^4^.

In this study, a watermarking model for brain images using LWT and SVM has been presented in Fig. 1^4^. In this case, a unique watermarking approach is employed, and to forecast where the watermark image will be inserted in the MR images, SVM is used. Double-layer security is included in our suggested model to guarantee the reliability of embedded data. A transform domain-based hybrid a watermarking approach is used to embed the scrambled data into the host image’s coefficients after the data has been obscured using a unique key. This suggested plan takes advantage of the secret key’s randomization characteristics and the reversibility of LWT.

The main contributions to this work are listed as follows:

- Region classification using SVM: The study employs support vector machine (SVM) to accurately distinguish between the region of interest (ROI) and non-region of interest (NROI) in brain images, ensuring that watermark embedding does not compromise critical diagnostic areas.
- Robust watermark embedding with LWT: By applying the lifting wavelet transform (LWT) to the NROI, the research achieves a robust embedding of watermark information, enhancing the scheme’s resilience against various attacks.
- Enhanced security via shared secret key: The introduction of a shared secret key adds an extra layer of security, bolstering the robustness of the watermarking process against unauthorized access and tampering.
- Comprehensive performance evaluation: The method is rigorously tested on brain image, with simulations performed to assess imperceptibility and robustness using various evaluation metrics (PSNR, NCC, SSIM).

This paper is organized as the following: In “Literature review” section, a survey of the literature has been completed. A few introductory details are described in “Support vector machine (SVM) classifier” section. In “Proposed watermarking system” section defines the developed watermark extraction and embedding algorithms. The experimental findings using the suggested framework in “The results of experiments” section. Finally, “Conclusion” concludes with some directions for further research.

## Literature review

Singh et al. published a watermarking method based on DWT-SVD^5^. The watermark is encrypted and encoded using elliptic curve cryptography (ECC). It increases the computational cost slightly but provides the resilience and imperceptibility of the suggested paradigm.

Roy et al.^6^ presented a novel steganographic system that makes use of the division of video frames technique. They use a region selection strategy in conjunction with principal component analysis (PCA), a dimensionality reduction procedure, to compress the areas and implant hidden data in such compact parts. By reducing the average square distance between the values of the pixels and those vectors, this PCA is used as a best-fit vector. According to the findings of their research, they might obtain better visual quality and increased embedding capabilities. Additionally, by enabling the recipient to retrieve the coded message even after specific authorized route invasions, the proposed method increases robustness. In 2021, Ji and Cheng created a monogenic feature-based adaptive multisensory picture fusion technique^7^. To achieve adaptive fusion, the suggested approach makes use of the monogenic characteristics present in the source images, separates them into distinct sections according to various similarity metrics, and selects various fusion algorithms. Singh et al.^8^ presented a state-of-the-art multi-focus fusion of images method for tracking in real time in 2021. The process of fusing images becomes more efficient by applying a multi-stage method, a wavelet-based hybrid solution, the noise diffusing and the principle the noise diffusing, In 2022, Cheng et al.^9^ suggested extracting and evaluating dermocopy pictures automatically using a Convolutional Neural Network architecture by detecting and evaluating troublesome skin regions. By 2022, Gaffar^10^ offered a special kind of visual steganography technique along with vulnerable embedded images or poor embedding capacity. The suggested approach, which is based on the golden ratio and the non-subsampled contourlet transform (GRNSCT) model, provides excellent picture secrecy and embedding capability.The huge embedding capacity is obtained via a two-level non-subsampled contourlet transformation, and anonymity is ensured by shuffle cards in a deck of cards method. The resilience of embedded images is examined using a variety of security evaluation measures, including information entropy, key sensitivity, and histogram. In 2021, Rasha^11^ looked into the use of medical picture watermarking for image honesty, assurance and authenticity. The present medical image validation techniques highlight the necessity of attaining resilience against inadvertent attacks (such as compressing and image noise), which cannot be avoided; hence, the current medical image validation techniques ought to confirm that they can resist these kinds of attacks. However, color medical images are not easily captured by the same medical image watermarking and medical image authentication plans as were designed for pictures that are just in black and white. This poses a new task for this field’s future research. Meng et al.^12^ introduced the unique data-obscuring method in 2022 that makes use of encrypted graphics according to IWT and chaotic systems. Using the IWT transformation, the carrier picture was divided into wavelet factors. Then, the chaotic system was used to create location, scrambling, and encryption phrases for data protection and image. The final encrypted image was created by employing the position sequence to conceal the concealed information in the diagonal element after the keys and scrambled processes were used to protect the approximate portion. When the wavelet transform factor became encrypted, the problem of pixel loss during the reconstructing process was resolved. The secret key that was required to unlock the photo and reveal the hidden message was split into two sections for varying degrees of security. The security and maximal payload of the solution were enhanced by the technique of encrypting images after data hiding. A novel polynomial decomposition-based image watermarking approach was proposed by Sabbane et al.^13^ in 2020. According to the experiment results, they were able to attain good perceptibility with an NCC value of 1 and a PSNR of 61.66 dB, Experimental results demonstrate that the proposed strategy achieves a decent balance of watermark invisibility and robustness when compared to state-of-the-art solutions. Zhang et al.^14^ examined a number of research issues with privacy of medical images in 2020. An and Liu^15^ presented a deep learning model-based approach for medical image segmentation in 2021. This study proposes a multi-level, boundary-aware RUNet segmentation model. The network structure consists of a multi-level border detection network and a segmenting system utilizing U-Net. It can find a solution to the border position problem. At the same time, our research suggests integrating new active self-awareness modules into deep learning models to solve the issue of the insufficient medical imaging adaptability of deep learning networks. A watermarking approach for medical images were proposed in the transform domain by Thanki et al.^16^ in 2017. They suggested using the DCT and the fast discrete curvelet transformation (FDCUT) as the foundation for a blind medical image watermarking method. To obtain the frequency coefficients of a medical image’s curvelet decomposition, FDCuT is applied. To obtain different frequency coefficients, the high frequency-curvelet coefficients of the medical image are extracted using block-wise DCT. To try to solve the security concern surrounding medical fundus photos for systematic computer-aided identification of retinal diseases and tele-ophthalmology uses, Singh et al.^17^ introduced an invisible watermarking system in 2017. A reversible data concealing approach for medical image applications was proposed by Parah^18^ in 2017. To guarantee the reversibility of medical images, during the creation of the cover image, a Pixel to Block (PTB) converter mechanism was used as an effective and cost-effective replacement for interpolation. For medical picture applications, they suggested a novel approach based on image interpolation^19^ in order to obtain a high payload data hiding system. Possessing a PSNR more than 35 dB and an SSIM higher than 0.50, Rai et al.^4^ improved robustness and imperceptibility in their 2017 watermarking system. A robust image watermarking approach was proposed by Ganic et al.^20^ in 2004. They encoded the data in all DWT and SVD frequency domains. Vulli et al.^21^ employed the 1-cycle approach in conjunction with the Fast AI framework in 2022. To determine and identify tumors using full slide pictures. Compared to other models already in use for the same purpose, they achieved higher accuracy. Yadav et al.^22^ created an effective stenographic strategy in 2023 for a secure communication framework in which data is hidden using a heuristic technique. To increase the method’s efficiency, they also employed a machine learning scheme. Garg et al.^23^ created a stenographic technique in 2022 that uses the neural style transfer (NST) method to embed the details contained in a hidden image within the hosting image’s styling data. Here, they optimize using discriminator loss to get 50% accuracy in the absence of noise and 35% accuracy in the presence of noise. A robust JPEG steganography based on SVD and DCT was proposed by Song et al.^24^ in 2022 in the no subsampled shear let transform domain, where they were able to attain improved anti-detection capabilities. In their contribution to the subject of safe data hiding, Hameed et al.^25^ presented a novel strategy that combines LSB substitution with an optimization method inspired by nature. The application of HHO enhances the security and imperceptibility of the steganographic process by resolving common problems with traditional LSB techniques. According Hassaballah et al.^26^, digital image steganography was a crucial field of research in information security that provided techniques for secret communication and data protection. The goal of ongoing research is to create increasingly complex methods that improve security, resilience, and capacity while tackling new issues with detection and defenses.

Hassaballah et al.^27^ combined ADPVD and HOG techniques to report a major breakthrough in color image steganography. Effective and undetectable data embedding is made possible by the method’s adaptive nature, which is led by local image attributes and edge directions. The HHO-IWT approach, which combines the Integer Wavelet Transform and the Harris Hawks Optimization algorithm, was proposed by Hassaballah et al.^28^. To encode hidden data in a way that minimizes distortion, the cover image is broken down into sub-bands using the Integer Wavelet Transform. To ensure that the steganographic method strikes a compromise between imperceptibility and payload capacity, the embedding process is optimized using the Harris Hawks Optimization algorithm. A notable development in image steganography was demonstrated by Hameed et al.^29^, who adaptively combined HOG with PVD and LSB techniques. The technique successfully made use of the properties of image content to improve data hiding capability, preserve visual quality, and offer resilience against steganalysis attacks. A breakthrough in image steganography was demonstrated by Abdel Hameed et al.^30^, who included adaptive directional analysis into the pixel value differencing framework. The ADPVD technique provides a strong solution for safe data concealing in digital images by skillfully balancing the trade-off between embedding capacity and imperceptibility. An adaptive visibility improvement method that includes Laplace filtering and bright balance was proposed by Bekhet et al.^31^. The technique successfully improves image clarity while maintaining color naturalness by adapting to different degrees of dust distortion. This flexibility guarantees that the method will continue to work under a variety of dusty circumstances. In the area of image processing for traffic applications in inclement weather, Kenk et al.^32^ made a noteworthy breakthrough. The authors provided a system that improves visibility in dusty situations while maintaining color fidelity by developing an adaptive technique that combines bright balance with Laplace filtering. This technology shows potential for increasing the dependability and safety of intelligent transportation systems operating in places prone to dust storms. By presenting an adaptive PVD-based technique that improved data hiding capability while maintaining image quality, Hassaballah et al.^33^ made a contribution to the field of image steganography. The drawbacks of earlier PVD techniques are addressed by the creative use of an adaptable range table, which enables effective and undetectable embedding.

By presenting an adaptive PVD-based technique that improved data hiding capability while maintaining image quality, Hassaballah et al.^33^ made a contribution to the field of image steganography. The drawbacks of earlier PVD techniques are addressed by the creative use of an adaptable range table, which enables effective and undetectable embedding. Future research could examine the application of this method to continuous action spaces and its integration with other RL frameworks to further enhance its applicability and robustness. Hafiz et al.^34^ showed a significant advancement in reinforcement learning by introducing an ensemble of binary action deep Q-networks. The approach successfully strikes a balance between simplicity and performance, providing a scalable solution for complex RL tasks. By combining 3-D bit-level replacement and multilayer differentiation techniques with a 9-D chaotic system, Mohamed Abdel Hameed et al.^35^ introduced a novel method for encrypting color images. By resolving the shortcomings of earlier lower-dimensional chaotic encryption techniques, the suggested approach seeks to offer a high degree of security while preserving the quality of the encrypted images. By putting forth a technique that strikes a compromise between improved security measures and great image quality, Hameed et al.^36^ made a substantial contribution to the field of medical image steganography. This method ensures that patient information is kept private and undamaged during online contacts, addressing the crucial necessity for secure transfer of medical images in telemedicine.

To fine-tune watermarking parameters, Awasthi et al.^37^ presented optimization approaches such genetic approaches (GA), particle swarm optimization (PSO), and grey wolf optimization (GWO). These methods reduce distortions and improve resilience in medical photographs. The combination of watermarking and ANFIS optimization allows for adaptive learning-based enhancements, which makes it a viable strategy for practical medical applications. Awasthi et al.^38^ presented a watermarking system that uses a denoising convolutional neural network (DnCNN) for image denoising, the Henon map for secure transmission, and fuzzy logic to determine the scaling factor for embedding. The goal of this method is to create an invisible watermarking mechanism that is resilient to several types of attacks. Algorithms for optimization have also proven essential in improving watermarking methods. A new bio-inspired meta-heuristic called the White Shark Optimization method has been used to find the best scaling parameters for watermark embedding, optimizing the trade-off between resilience and imperceptibility. When Braik et al.^39^ first presented this technique, they showed how effective it is at solving global optimization issues. This approach can be used for watermarking medical images. Awasthi et al.^40^ employed the Advanced Encryption Standard (AES) for the initial authentication layer, ensuring the verification of the patient’s identity. For secondary authentication, the system utilizes binary robust invariant scalable key points (BRISK) and minimum eigenvalue (MinEigen) features to authenticate watermarked images. This dual-layer approach aims to prevent forgery and unauthorized access effectively. Tiwari et al.^41^ offers a watermarking strategy that integrates the lifting wavelet transform (LWT), discrete cosine transform (DCT), and singular value decomposition (SVD). The process creates reference sub-images for watermark extraction and embedding by using the Canny edge detector to find high-edge blocks in an image. Several scaling factors, which are optimized using particle swarm optimization (PSO), modulate singular value coefficients during embedding to strike a balance between robustness and imperceptibility. Results from experiments show improved performance over current schemes and increased resilience to different types of attacks. Table 1 gives a lot of literature reviews about digital watermarking techniques.

**Table 1:** Some of literature reviews about of digital watermarking techniques.

Paper/study	Authors	Year	Key contributions	Limitations
Effective steganographic strategy for secure communication using a heuristic technique	Yadav et al.^22^	2023	Suggested steganographic architecture based on heuristics and used machine learning to increase efficiency Improved concealed data security and resilience	Heuristic methods might not be generalizable Higher computational overhead because of integrating ML
Steganographic technique using neural style transfer (NST) for image embedding	Garg et al.^23^	2022	Hidden images were embedded into hosted photos using NST. Discriminator loss optimization was used to increase accuracy Accuracy was 35% with noise and 50% without	Reduced precision in noisy environments Adversarial attacks against NST-based embedding are a possibility
Adaptive PVD-based technique for data hiding	Hassaballah et al.^33^	2018	Enhanced ability to conceal data without sacrificing image quality Increased resistance to attacks using JPEG compression (range 10–90)	For various kinds of images, it might need to be adjusted Limited comparison with sophisticated steganalysis techniques
Robust JPEG steganography using SVD and DCT in shear let transform domain	Song et al.^24^	2022	Employed the discrete cosine transform (DCT) and singular value decomposition (SVD) in the non-subsampled shear let domain Improved anti-detection functionality Increased resistance to steganalysis	Complex transformations result in a higher computing cost Robustness and embedding capacity are traded off
ANFIS optimization-based watermarking for securing integrity of medical images with blockchain authentication	Awasthi et al.^37^	2024	Watermark optimization was done using the adaptive neuro-fuzzy inference system (ANFIS) Used blockchain-based authentication to guarantee data integrity in medical photographs Enhanced resistance to geometric and noise threats	ANFIS requires a large amount of training data The inclusion of blockchain raises processing and storage requirements

### Limitations of existing works

Watermarking, steganography, image fusion, and medical imaging have all benefited greatly from the contributions reviewed studies’ contributions, but there are still several issues that need to be resolved like:

- Computational complexity: Many methods, including deep learning-based models (Ji and Cheng^7^), and Singh et al.’s^5^ ECC-based watermarking, require a lot of processing power, which makes them inappropriate for real-time applications.
- Attack vulnerability: Robustness and dependability are impacted by several approaches, such as those based on PCA compression and DWT-SVD, which are susceptible to geometric distortions, noise, and powerful compression attacks.
- Trade-off between security and imperceptibility: While embedding capability is given priority in techniques like GRNSCT-based steganography and NST-based stenography, imperceptibility may be compromised by obvious distortions.
- Limited generalizability: The usefulness of many deep learning-based techniques, such as those suggested for medical image analysis, is limited by their high training data requirements and dataset bias.
- Absence of real-world testing: Some suggested techniques have only been tested in controlled settings without being widely used in the real world, which raises questions regarding their applicability and efficacy in various scenarios.
- Storage and bandwidth restrictions: Some approaches, particularly those that use complicated steganographic techniques or multi-stage image fusion, may result in higher storage needs and processing delays, which can be problematic in contexts with limited bandwidth.
- Dependency on parameter tweaking: Several encryption and fusion methods necessitate manual parameter tweaking, which limits automation and flexibility to accommodate various picture formats and application requirements.

## Support vector machine (SVM) classifier

To embed the watermark in the non-essential component of a brain image, it is necessary to classify the ROI and NROI regions. However, by using SVM as a classifier, this procedure can be prevented by avoiding distortion of the diagnosis area of the image. Thus, the schematic watermarking methods can be made easier with the help of such a classification strategy. An SVM and SS-based watermarking system was proposed by Ramly et al.^42^. Spread spectrum has been used to embed and extract patient data, while SVM has been used to classify NROI and ROI in brain images. Nonetheless, the suggested model has not been the target of any attacks; instead, embedding has taken place in the spatial domain. A new method of watermarking employing a supervised machine learning algorithm was presented by Yen and Wang^42^. Given that the embedding was completed in the spatial domain, which is amenable to a variety of image processing methods, the watermark is added by asymmetrically adjusting the blue channels of the core and surrounding pixels.

## Lifting wavelet transform (LWT)

**Fig. 2:**
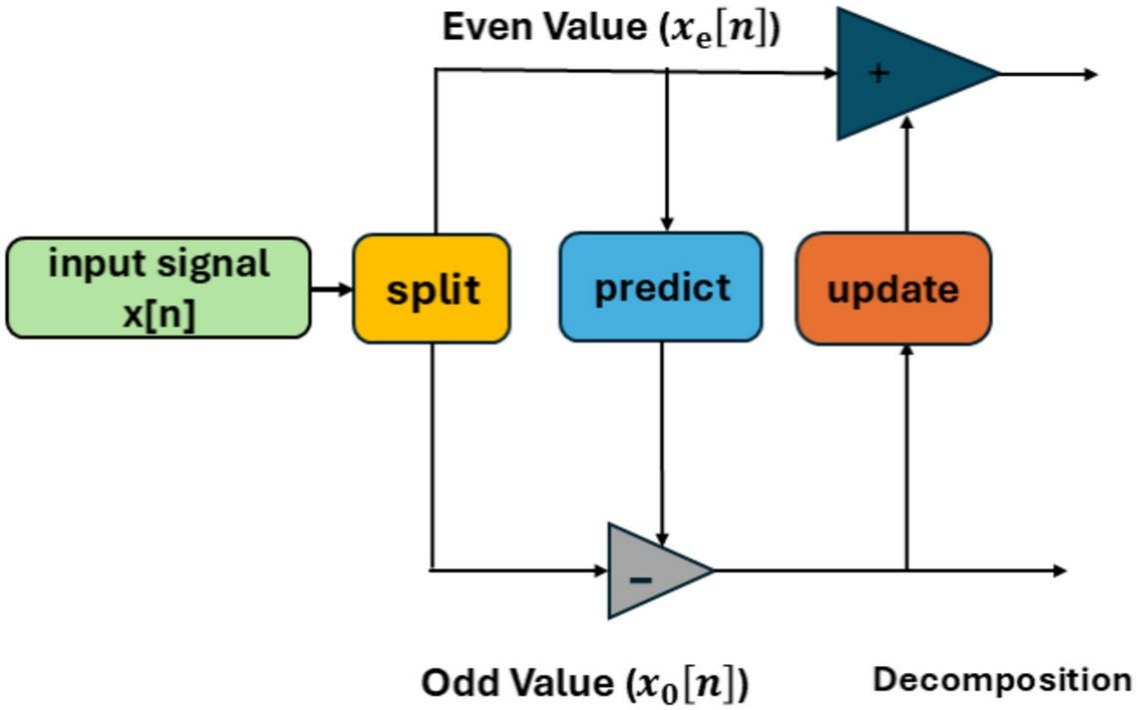
Block schematic of the lifting wavelet transform^52^.

Since the LWT may be implemented more quickly and effectively than the conventional wavelet transforms^43^, it has gained significant traction as a potent tool for image analysis in recent years, according to Sweldens^44^. In the areas of image de-noising^45^, image compression^46^, feature extraction^47^, pattern recognition^48^, and watermarking^49,50^, it performs better than standard wavelets. Sweldens^43^ and Daubechies and Sweldens^51^ provide a thorough mathematical explanation of the lifting strategy. The lifting-based wavelet transformations address the drawbacks of conventional wavelets with their improved frequency localization feature and time savings. The primary concept underlying lifting wavelets is to take a basic wavelet shape and use it to produce a new wavelet with better qualities. The transformation of lifting wavelets is an alternative to discrete wavelet transformation. The second-generation wavelets are constructed using a lifting technique, and translations and expansions of an individual function are occasionally performed. Assembling wavelets with a lifting method involves three steps: Split, Predict, and update. Data is divided into odd and even sets during the split phase. In the predict stage, an odd set is predicted using an even set. In high pass, polynomial cancellation is guaranteed by the predicted phase. Wavelet coefficients will be used in the update phase to compute the scaling function and modernize the even set. The lifting wavelet transform approach is shown in Fig. 2^52^. The wavelet transform can be implemented more quickly thanks to the lifting approach.

**Fig. 3:**
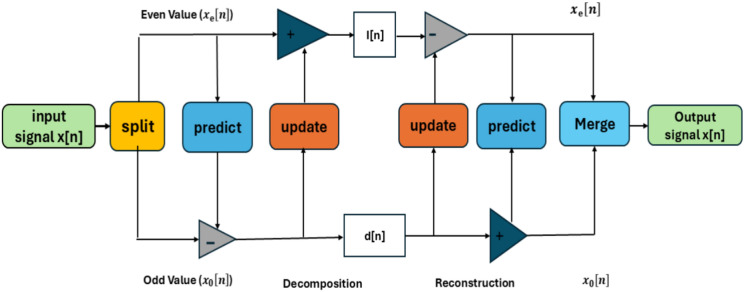
Signal degradation in lifting wavelet transformation^52^.

The three processes of signal decomposition using LWT are splitting, predicting, and updating. These steps are seen in Fig. 3^52^ and are explained as follows:

Split the original signal x[n] into odd and even samples that do not overlap, or $x_{e}$[n] for even samples and $x_{0}$[n] for odd samples, where^52^

1 $$x_{e} \left[ {\text{n}} \right] = {\text{x}}\left[ {2{\text{n}}} \right],\;\;x_{{0{ }}} \left[ n \right] = x\left[ {2n + 1} \right]$$

Assume that even and odd samples have a correlation so that one can serve as the other’s predictor.

${\text{x}}_{0}$[n] the following samples are utilize ${\text{x}}_{{\text{e}}}$[n] to predict:

2 $${\text{d}}\left[ {\text{n}} \right] = x_{{0{ }}} \left[ n \right] - p\left( {x_{e} { }\left[ n \right]} \right)$$

where P(·) is the predictor operator and d[n] are the difference between the original sample and its predicted value, which is defined as a high-frequency component.

With the aid of updates function U(·) and description signal d[n], updates might be made to the even sampling. The original signal’s approximate form is then represented by the elements with low frequency I[n], which are then derived as follows^52^:

3 $${\text{I}}\left[ {\text{n}} \right] = x_{e } \left[ n \right] + U\left( {{\text{d}}\left[ {\text{n}} \right]} \right)$$

## Proposed watermarking system

### Image classification

In this classification stage of the proposed model, the pixels of the medical host image are categorized into ROI and NROI to do embedding. The labeled predictor data in an SVM classification model is utilized to create a prepared model. It is possible to use several SVM kernel procedures to find the right predictive precision, which means that changing certain parameters will result in higher accuracy. The test data set is used for cross-validation of the model after it has been trained. For example, NROI and ROI are the two divisions of the host brain image. To control its associated parameters, the training SVM model uses the provided dataset to incorporate features such as the bias and weight vector; they are mostly utilized to minimize the misclassification among NROI and ROI. While NROI isn’t as important, ROI is primarily utilized for diagnostic purposes. Consequently, even a minor error in the ROI and NROI selection process might have a significant negative impact on diagnosis.

### Embedding a watermark

**Fig. 4:**
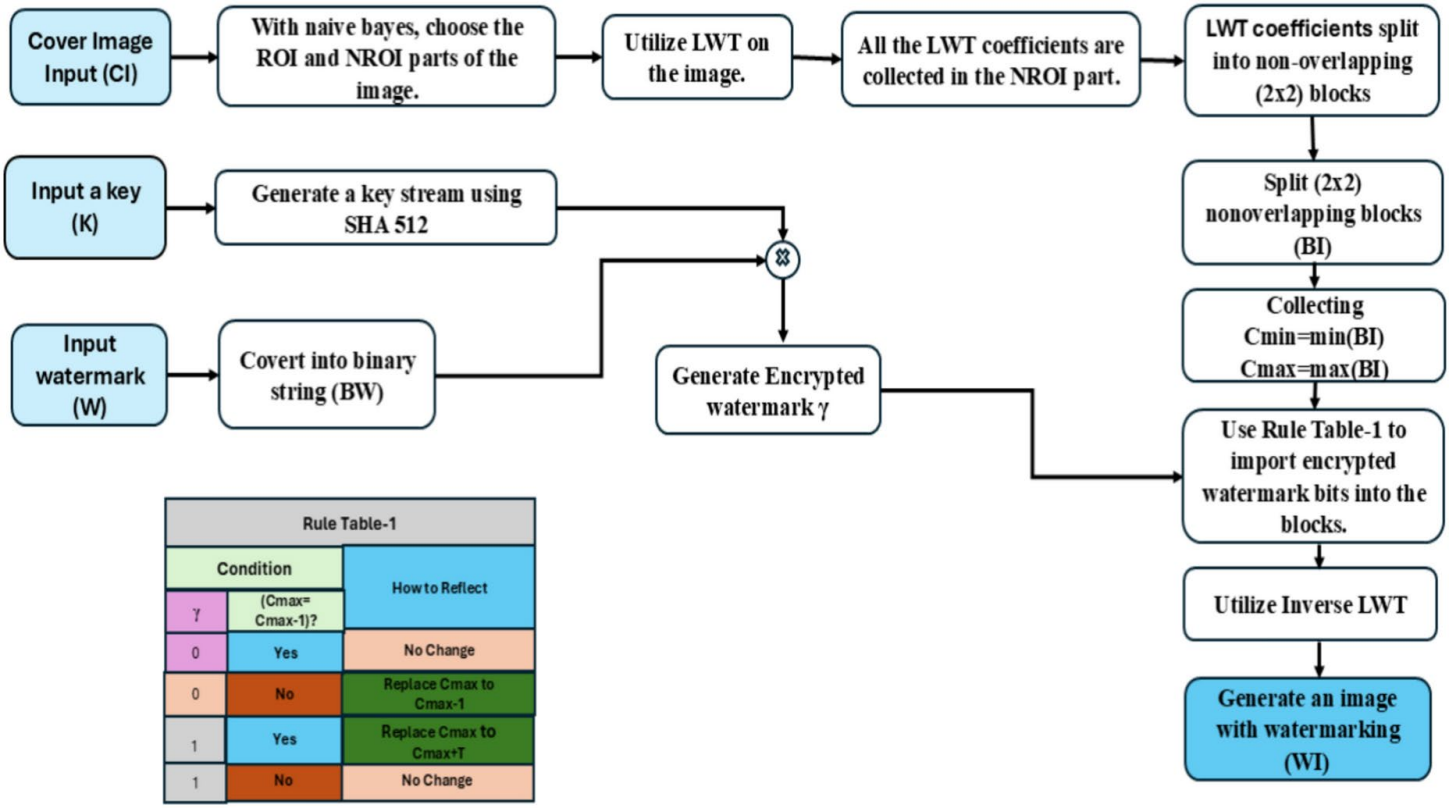
Proposed watermarking scheme block diagram.

A description of the embedding scheme of the proposed scheme is contained in this section. Figure 4 describes the fundamental block diagram of the suggested architecture. An input image of size (M × N) is initially used. The use of SVM to choose the NROI and ROI portions of an image has already been covered, as was done in the previous section. Now, the pixel values for these NROI and ROI places are kept in two distinct arrays. Let’s title them $\upbeta _{r}$ and $\upbeta _{{{\text{nr}}}}$. The two further arrays, $\upalpha _{r}$ and $\upalpha _{nr}$, contain the location coordinates of the pixels. In this instance, only the ROI area of the image is considered important. Collect every LWT coefficient found in the NROI region of the images after applying lifting wavelet transform (LWT) to them. Now, choose any unique secret key (κ) and use the common SHA-512 algorithm for encryption to create a 512-bit string. Thus, create the watermarked bit stream (BW) and input the watermarked logo (W). Therefore, to create an encrypted watermark (γ), K and the BW are XORed. Using the rule table-1 provided in Fig. 4, the (γ) has now been incorporated into the cover image’s NROI area.

Here, T can be selected at random from each block by applying the following formula: T = $C_{{{\varvec{min}}}}$ mod 8. Essentially, the watermark bit is jumbled using a shared secret key. For every iteration, four pixels of the cover image contain four bits of watermark information that has been encoded. The additional NROI spots will follow this procedure. Once all the γ bits have been incorporated into the cover image, create the watermarked image (WI) by using inverse LWT. Algorithm 1 has supplied the embedding algorithmic framework.

## Extracting watermark

**Fig. 5:**
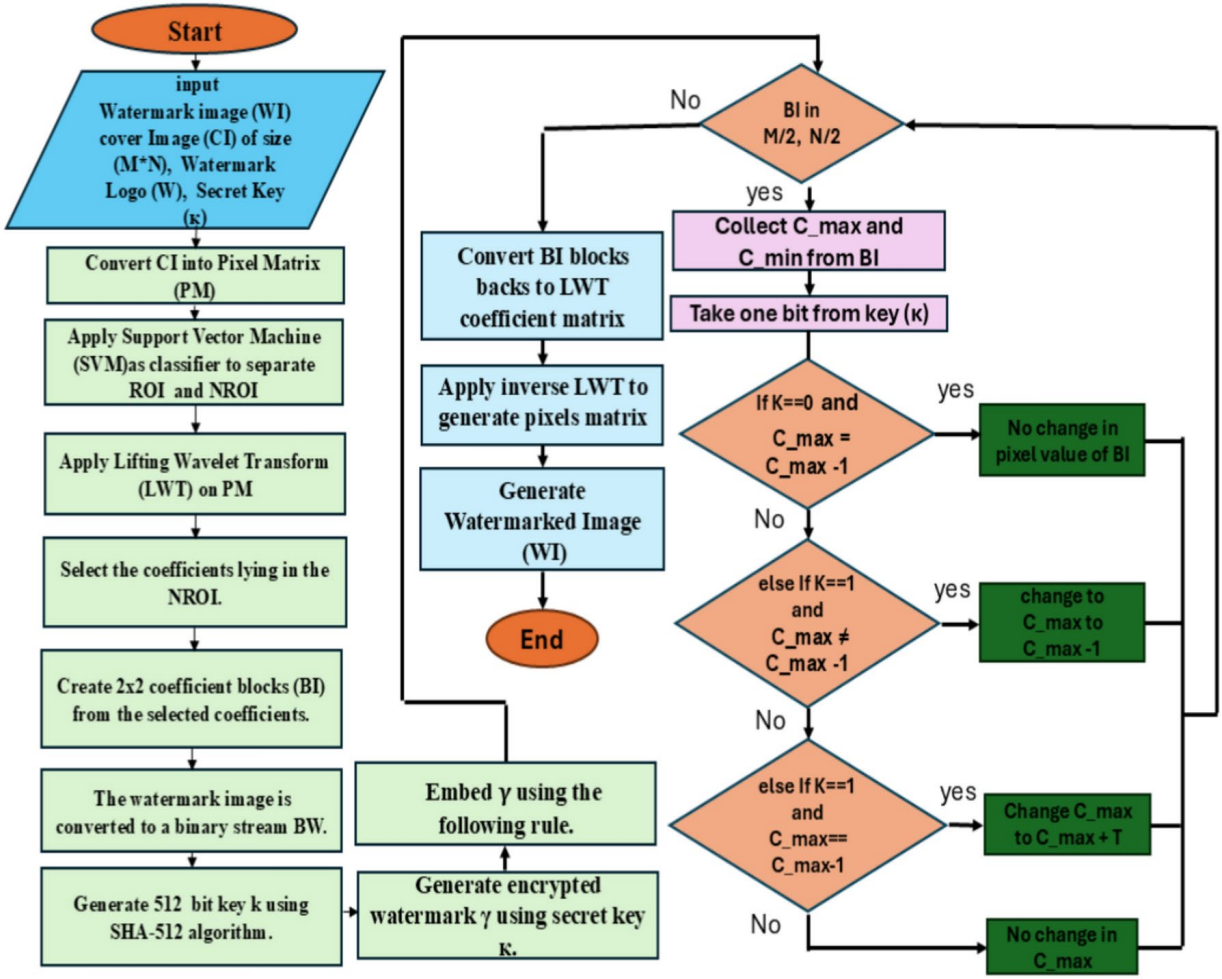
The flowchart of embedding watermark.

This section has provided a description of the watermark information extraction procedure. The first input to be considered is the size (M × N) watermarked image (WI). The NROI and ROI regions of the image with the watermarking has been chosen utilizing SVM in accordance with “Support vector machine (SVM) classifier” section rules. Now, the pixel values for these NROI and ROI are saved in two distinct arrays, let’s say $\upbeta _{r}^{\prime}$ and $\upbeta _{nr}^{\prime},$ the locations of pixels are recorded in two other arrays, let’s say $\upalpha _{r}^{\prime}$ and $\upalpha _{nr}^{\prime}$ given that the watermark data was only incorporated into the images’ NROI region, this is the only area of the photographs that is of concern. The watermarked image is now subject/ed to the lifting wavelet transform (LWT), from which the LWT coefficients are extracted. Divide The values of coefficients into 2 × 2 not overlap blocks, then take a value of $C_{max}$ and $C_{min}$ from each block. Therefore, compare $C_{max}$ and ($C_{max}$ − 1) and extract 0 if they are the same; if not, extract 1 and store them in an array $\upgamma^{\prime}$. Like an encrypted watermark bit is this $\upgamma^{\prime}$. Retrieve 1 and place them in an array $\upgamma^{\prime}$. An encrypted watermark bit is what this $\upgamma^{\prime}$ represents. Use the standard SHA 512 encryption technique to create a 512-bit string after choosing the shared secret key (κ). Continue by XORing $\upgamma^{\prime}$ with κ to obtain the original watermark segments. From the watermark information, the watermark logo may be produced with ease. The following flowchart is used for embedding watermarking (Fig. 5). This flowchart embeds information into the image’s less noticeable portions (NROI) to provide safe, reliable, and undetectable watermarking. The LWT transformation makes sure that changes don’t have a big effect on the quality of the images. Unauthorized access is prevented via the SHA-512 encryption algorithm.

**Fig. 6:**
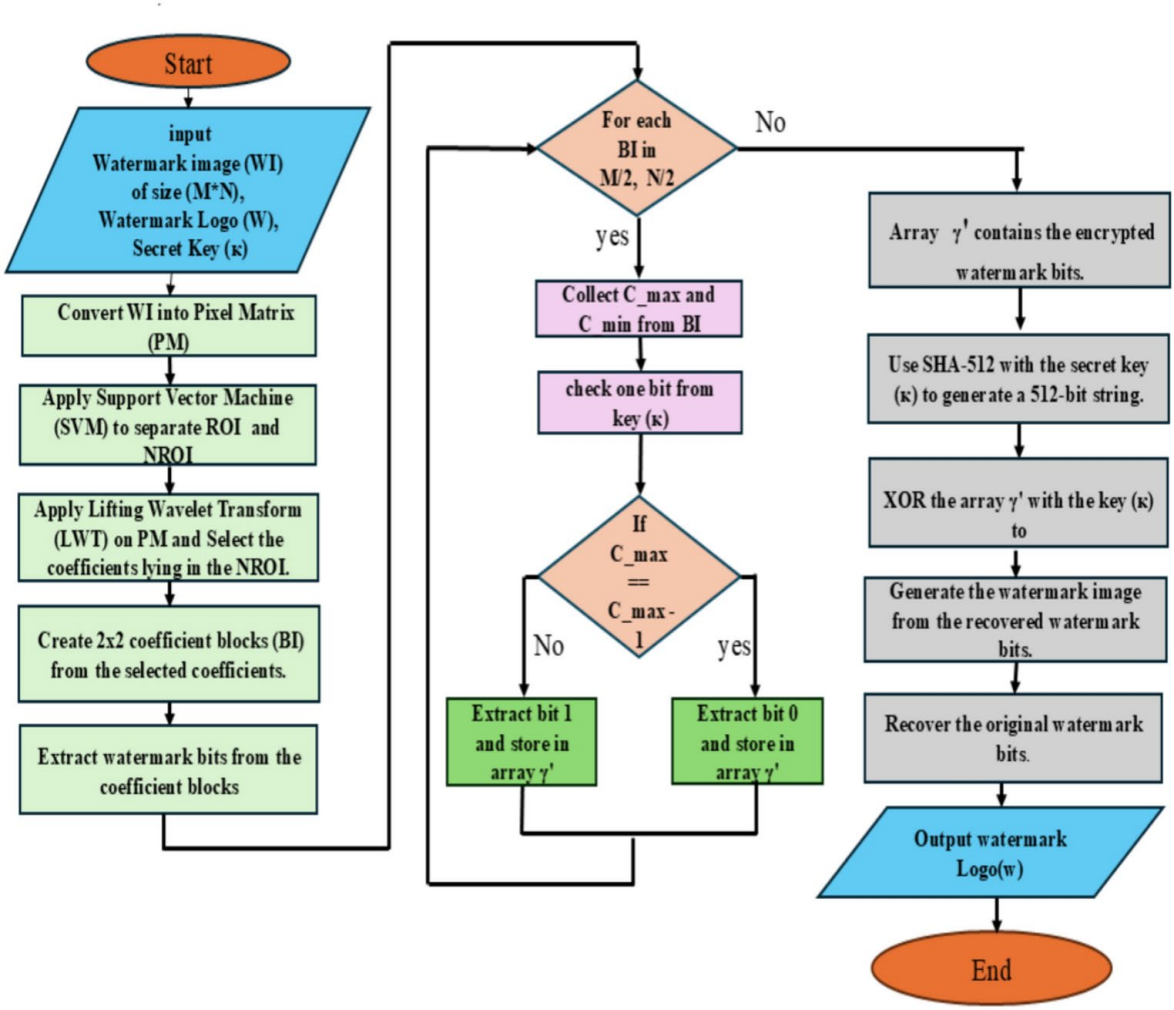
The Flowchart of extraction watermark.

**Fig. 7:**
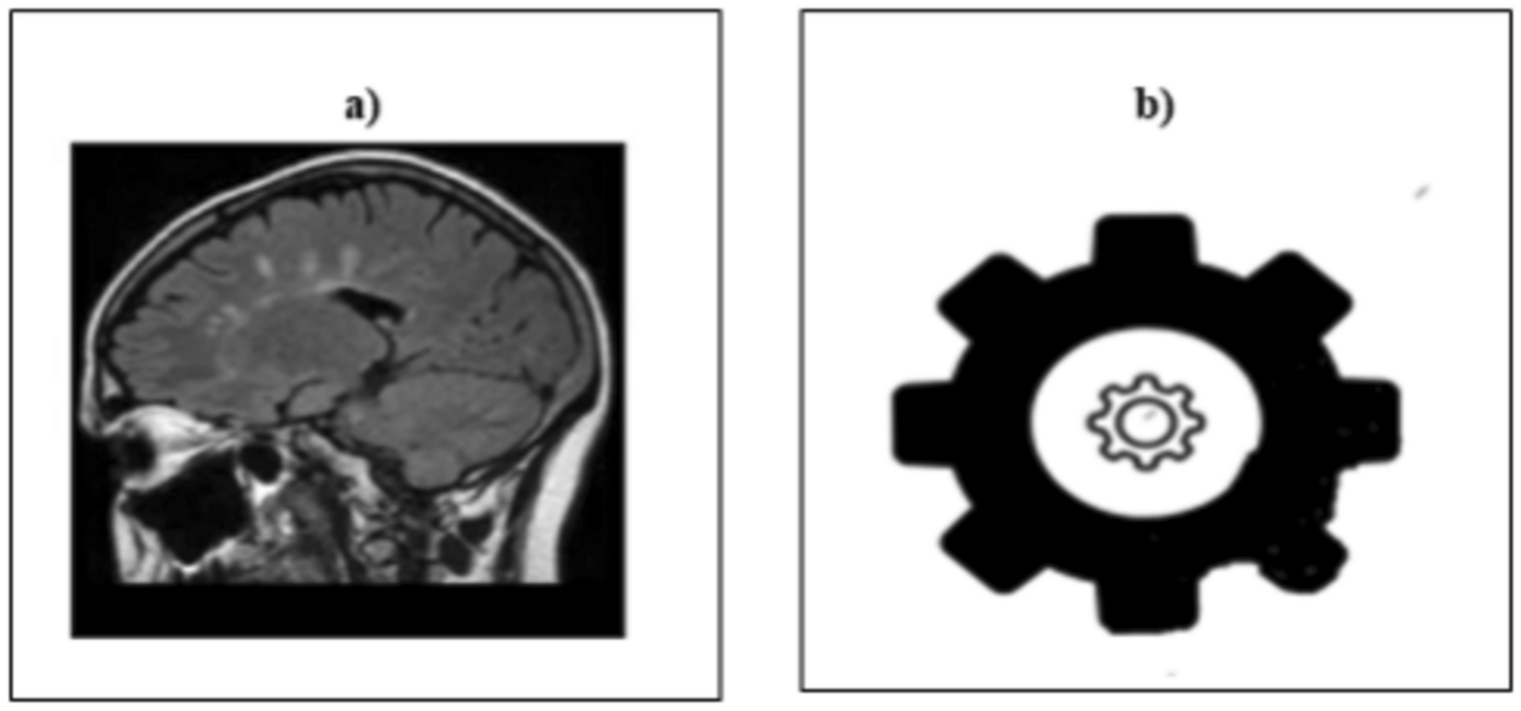
(**a**) Watermarked brain image (**b**) Extracted watermark image.

The information gathered from the watermark allows us to quickly confirm or verify the cover sheet. Figure 6 provides the extraction’s watermarking basis. Finally, Fig. 7 provided watermarked brain image and extracted watermark image.

## The results of experiments

The experiment makes use of an Intel Core i7, 2.26 GHz CPU, Microsoft Windows 10 operating system, a 500 GB HDD and 16 GB of DDR3 memory. The suggested model is tested on a collection of One hundred and fifty brain image—256 × 256 JPEG grayscale images. The experiment’s database is sourced from MATLAB Central (brain images) Reference^4^. The 128 × 128 grayscale image used as a watermark is displayed with each 256 × 256 JPEG image in the experiment; there are 65,536 predictor data points in total. The SVM model uses these data points, which are further divided into two areas known as training data and testing data.

Of the total data provided, 6553 data points, or 10%, are used for training, and the remaining 58,983 data points, or 90%, are used to cross-validate the learned classification model. The input image’s intensity values and pixel locations are the feature values utilized here for classification; the right threshold value is utilized to select the features, and the outcome is visible to the unaided eye. The trained system utilized parameters to categorize an image into ROI and NROI. The system is validated utilizing a test set of data after it has been trained. The experiment’s outcome shows that the suggested categorization model’s precision is 99.90%.

The suggested model’s resilience with varying watermark sizes is evaluated using the SSIM value. As the watermark’s size decreases, the matching SSIM value rises. Consequently, this conclusion demonstrates that the image’s resilience is dependent on the watermark’s size, with robustness increasing as the watermark’s size lowers.

There is another important factor in watermarking brain images which should be taken into consideration when assessing the total efficiency of the suggested strategy, which are.

### Capacity

Another important factor in watermarking brain images is capacity, which should be taken into consideration when assessing the total efficacy of the suggested strategy. It is the bit-per-pixel (BPP) value representing the total amount of information encoding in the host image. Equation (4) can be used to compute the capacity^52^.

4 $$Capacity = \frac{{Total\;number\;of\;bits\;that\;can\;be\;embeded{ }}}{Total\;number\;of\;pixels\;in\;the\;image}\;Bpp$$

### Measures of visual quality

A watermarking system’s visual quality is assessed using the performance measures listed below.

#### PSNR (peak signal to noise ratio)

To evaluate the visual degradation created by enclosing the hidden image inside the cover image, one uses the PSNR^52^:

5 $$PSNR = 10\log_{10} \frac{{255^{2} }}{MSE}$$

The definition of the mean square error (MSE) is as follows^52^:

6 $$MSE = \mathop \sum \limits_{i = 1}^{m} \mathop \sum \limits_{i = 1}^{n} \frac{{I\left( {i,j} \right) - SI\left( {i,j} \right)}}{m \times n}$$

where I (i, j) and SI (i, j) represent the pixel intensities of the original and watermarked images with sizes of (m × n), respectively.

#### Measure of structural similarity index (SSIM)

To assess how well the watermarked image (SI) and host image (I) can be differentiated from one another, SSIM is applied in addition to PSNR. Three factors are compared by SSIM to determine how similar two images are: brightness, structure and contrast. Equation (7) provides a definition of the SSIM of I and SI^52^.

7 $$SSIM\left( {I,SI} \right) = \alpha \left( {I,SI} \right){\boldsymbol{\beta}}(\left( {I,SI} \right){\boldsymbol{\gamma}}(\left( {I,SI} \right)$$

were

8 $$\alpha \left( {I,SI} \right) = \frac{{2\delta_{1} \delta_{{SI{ }}} + c_{1} }}{{\delta_{1}^{2} + \delta_{SI}^{2} + c_{1} }}$$

9 $${\boldsymbol{ \beta }}\left( {I,SI} \right) = \frac{{2\delta \alpha_{1} \delta \alpha_{{SI{ }}} + c_{2} }}{{\delta \alpha_{1}^{2} + \delta \alpha_{SI}^{2} + c_{2} }}$$

10 $${\boldsymbol{\gamma}}\left( {I,SI} \right) = \frac{{\sigma_{I.SI} + c_{3} }}{{\sigma_{I} + \sigma_{SI} + c_{3} }}$$

Here, the brightness of the corresponding images is represented by $\updelta _{{\text{I}}}$ and $\updelta _{{{\text{SI}}}}$. The greatest value of α is one if the luminance of both images is the same, that is, $\updelta _{{\text{I}}}$ = $\updelta _{{{\text{SI}}}}$. In a similar vein, the highest value of β is also 1 if the contrast of both images is the same, but in structural comparison, The correlated factors between the watermarking and host images are measured using γ. In this case, the covariance factors of the host and watermarked images are represented by $\upsigma _{{{\text{I}}.{\text{SI }}}}$, and the standard deviation is represented by $\upsigma _{{\text{I}}}$ and $\upsigma _{{{\text{SI}}}}$.

#### Normalized correlation coefficients (NCC)

A statistic called normalized correlation coefficients (NCC) is used to evaluate how resilient the watermarking system is. The correlation coefficients between the originally placed watermark (W) and the one that is retrieved (W) are computed; Eq. (11) is given by^52^:

11 $$NCC = \frac{{\mathop \sum \nolimits_{i = 1}^{m} \mathop \sum \nolimits_{j = 1}^{n} W^{\prime}\left( {i,j} \right) - W\left( {i,j} \right)}}{{\sqrt {\mathop \sum \nolimits_{i = 1}^{m} \mathop \sum \nolimits_{j = 1}^{n} W\left( {i,j} \right)^{2} \sqrt {\mathop \sum \nolimits_{i = 1}^{m} \mathop \sum \nolimits_{j = 1}^{n} W{^{\prime}}\left( {i,j} \right)^{2} { }} } }}$$

The ideal value for the NCC value is unity, and it is specified on intervals 0 and 1.

The size of the cover image under consideration is 256 × 256, meaning that 65,536 data points in total were employed for the categorization.

The experimental findings for samples of brain images for PSNR, SSIM, Capacity and NCC are shown in Table 2. An image that has a PSNR value above 30 dB is regarded as being of good quality for any typical image processing experiment. Table 8 makes it evident that the suggested scheme’s PSNR is roughly 64 dB. The human visual system (HVS) is unable to recognize the watermarked image.

**Table 2:** The experimental results of PSNR, SSIM, capacity and NCC.

Image	PSNR	SSIM	Capacity	NCC
brain_001.jpg	67.78	0.9999	63,527	0.9999
brain_002.jpg	67.86	0.9999	65,524	0.9999
brain_003.jpg	67.97	0.9999	63,690	0.9999
brain_004.jpg	67.99	0.9999	61,683	0.9999
brain_005.jpg	67.59	0.9999	65,689	0.9999
brain_006.jpg	67.88	0.9999	62,751	0.9999
brain_007.jpg	67.78	0.9999	65,476	0.9999
brain_008.jpg	67.78	0.9999	64,489	0.9999
brain_009.jpg	67.51	0.9999	65,355	0.9999
brain_010.jpg	67.51	0.9999	65,345	0.9999
brain_011.jpg	67.85	0.9999	64,662	0.9999
brain_012.jpg	67.66	0.9999	64,589	0.9999
brain_013.jpg	67.79	0.9999	65,455	0.9999
brain_014.jpg	67.89	0.9999	65,645	0.9999
brain_015.jpg	67.95	0.9999	63,751	0.9999
brain_016.jpg	67.97	0.9999	64,590	0.9999
brain_017.jpg	67.88	0.9999	63,683	0.9999
brain_018.jpg	67.88	0.9999	62,651	0.9999
brain_019.jpg	67.85	0.9999	65,472	0.9999
brain_020.jpg	67.98	0.9999	64,793	0.9999

Furthermore, it has been found that the experimental results on NCC and SSIM validate the robustness of the proposed method. The average NCC and SSIM findings for the twenty images are roughly 99% and 98%, correspondingly; this is sufficient to demonstrate robustness. A good trade-off between robustness, capacity, embedding, and imperceptibility is offered by the suggested method.

Tables 3, 4, 5, 6 and 7 compares the suggested strategy with a few state-of-the-art techniques currently in use. PSNR, Capacity, NCC, Time complexity and SSIM serve as the basis for the comparison. Table makes it evident that the suggested scheme’s PSNR is higher than that of the approaches mentioned.

**Table 3:** Comparison of the PSNR proposed scheme.

Method	Average PSNR (dB)
Abdel-Aziz et al.^53^	53.52
Moya-Albor et al.^54^	53.8638
Pulgam et al.^55^	30.304 (Ranged from 24.74 to 36.07)
Mishra et al.^56^	51.90 dB
Singh et al.^57^	41.03
Kiran et al.^58^	32.76
Swaraja et al.^59^	35.68
Jeevitha et al.^60^	49.536
Zarrabi et al.^61^	50.69375
Rai et al.^4^	52.18
Proposed system	67.81

**Table 4:** Comparison of the SSIM proposed scheme.

Method	SSIM
Abdel-Aziz et al.^53^	0.9998
Moya-Albor et al.^54^	0.9993
Pulgam et al.^55^	0.92111 Ranged from (0.81 to 0.97)
Mishra et al.^56^	0.9991
Singh et al.^57^	0.99
Kiran et al.^58^	0.4562
Swaraja et al.^59^	**–**
Jeevitha et al.^60^	**–**
Zarrabi et al.^61^	**–**
Rai et al.^4^	0.9872
Proposed system	0.9999

**Table 5:** Comparison of the NCC proposed scheme.

Method	NCC
Abdel-Aziz et al.^53^	1
Moya-Albor et al.^54^	0.9937
Pulgam et al.^55^	Ranged (0.8846 to 0.9999)
Mishra et al.^56^	1
Singh et al.^57^	0.99
Kiran et al.^58^	–
Swaraja et al.^59^	0.98
Jeevitha et al.^60^	–
Zarrabi et al.^61^	–
Rai et al.^4^	–
Proposed system	0.9999

**Table 6:** Comparison of the time complexity proposed scheme.

Method	Embedding time	Encryption time	Extraction time	Decryption time
Abdel-Aziz et al.^53^	–			
Moya-Albor et al.^54^				
Pulgam et al.^55^	0.0349 s	–		
Mishra et al	–			
Singh et al.^57^	0.88 s	0.83 s		
Kiran et al.^58^	–	0.1225 s	–	–
Swaraja et al.^59^	–	–	–	–
Jeevitha et al.^60^	–	–	–	–
Zarrabi et al.^61^	0.67061 s	0.78226 s		
Rai et al.^4^	–	–	–	–
Proposed system	3.4 s and 3.2 s		2.87 s and 2.77 s

**Table 7:** Comparison of the capacity proposed scheme.

Method	Capacity
Abdel-Aziz et al.^53^	–
Moya-Albor et al.^54^	329,960 bits (max)
Pulgam et al.^55^	–
Mishra et al.^56^	5120 bits
Singh et al.^57^	2 bPP
Kiran et al.^58^	–
Swaraja et al.^59^	925,184 bits
Jeevitha et al.^60^	–
Zarrabi et al.^61^	1.5 bPP
Rai et al.^4^	–
Proposed system	65,689 bits (max)

## Analysis of multi-criteria decisions

The PSNR variation under different scaling factors is shown in Table 8. One hundred and fifty images with increasing factors of 0.01, 0.02, 0.04 and 0.08 were taken into consideration. The original image’s average PSNR is generally 67.81 dB. However, for the 0.01, 0.02, 0.04 and 0.08 scaling factors, the average PSNR values drop to 64.56 dB, 57.58 dB, 49.1 dB and 42.33 dB, respectively. This indicates that the watermarked image may still be invisible to the human brain.

**Table 8:** Imperceptibility test results (PSNR).

Original image	Original	Different scaling factor of PSNR (dB)			
		0.01	0.02	0.04	0.08
brain_001.jpg	67.78	64.34	58.28	49.55	42.75
brain_002.jpg	67.86	64.67	57.69	48.86	41.96
brain_003.jpg	67.97	64.87	56.98	48.95	41.73
brain_004.jpg	67.99	64.56	58.55	49.95	42.97
brain_005.jpg	67.59	64.58	58.52	49.56	42.92
brain_006.jpg	67.88	64.83	57.52	48.95	41.84
brain_007.jpg	67.78	64.38	57.44	48.5	41.83
brain_008.jpg	67.78	64.79	57.52	48.63	41.58
brain_009.jpg	67.51	64.7	57.88	48.87	41.89
brain_010.jpg	67.51	64.9	57.89	48.64	41.56
brain_011.jpg	67.85	64.77	57.29	49.08	42.79
brain_012.jpg	67.66	64.59	56.63	48.59	41.98
brain_013.jpg	67.79	64.69	56.98	48.57	42.23
brain_014.jpg	67.89	64.88	57.64	49.96	42.89
brain_015.jpg	67.95	64.77	57.63	49.59	42.84
brain_016.jpg	67.97	64.56	57.4	48.98	42.96
brain_017.jpg	67.88	64.44	56.43	48.82	42.58
brain_018.jpg	67.88	62.55	57.61	49.95	42.56
brain_019.jpg	67.85	64.66	57.88	49.89	42.85
brain_020.jpg	67.98	64.72	57.89	48.99	41.98
Average	67.8175	64.5625	57.5825	49.144	42.3345

Additionally, the proposed technique was tested in a variety of attack situations, including rotation (90°), cropping, histogram equalization, filtering, JPEG compression, salt and pepper noise, and Gaussian noise. Table 9 presents the findings. The extracted watermark image’s PSNR value is consistently over 30 dB, as this table demonstrates. Excluding rotation and JPEG compression. This indicates that the HVS system can identify the watermark on the image with ease.

**Table 9:** Comparing results in various attack/noise scenarios.

SI#	Attacks/noise	PSNR	SSIM	NCC
1	Gaussian noise (SD = 1)	41.45	0.8297	0.7999
2	Impulsive noise (2%)	46.87	0.8839	0.8826
3	Salt and pepper noise (1%)	39.20	0.7835	0.8058
4	JPEG compression (Q = 40)	25.32	0.7798	0.7659
5	Speckle noise (1%)	36.37	0.7259	0.7938
6	Rotation (5°)	23.36	0.7598	0.7537
7	Histogram equalization	41.50	0.8294	0.7955
8	Brightness enhancement (25%)	44.33	0.8538	0.8730
9	Contrast enhancement (25%)	43.51	0.8455	0.8798
10	Log transformation (c = 0.1)	43.57	0.8619	0.8915
11	Average filtering (3 × 3block)	46.92	0.8769	0.8849
12	Circular average (R = 5)	44.29	0.8789	0.8859
13	Translation	45.52	0.8465	0.7869
14	Flipping (vertical)	41.59	0.7967	0.7967
15	Median filtering (4 × 4 block)	40.12	0.7992	0.7889
16	Cropping (10%)	39.78	0.7954	0.7994
17	Scaling + filtering	15.51	0.5534	0.5778
18	Flipping + shearing	14.23	0.4655	0.4739
19	Sharpen + salt and paper noise	17.29	0.5954	0.5963
20	Scaling (10%)	29.94	0.7734	0.7838
21	Shearing (X-shearing by 1.3)	22.20	0.6466	0.6799
22	Affine transformation	27.90	0.7596	0.7849
23	Rotation + contrast	15.33	0.4525	0.4658

When the suggested strategy is implemented with MATLAB, respectively, the mean embedding time is 3.4 s and 3.2 s, and the mean time needed for extraction is 2.87 s and 2.77 s (Table 9).

## Conclusion

In the healthcare sector, diagnostic data or medical images are transferred between locations via wired or wireless media. Thus, greater security is needed for the transfer of such data. The suggested paradigm guarantees that brain images are imperceptible and robust against attacks such as Gaussian noise, JPEG compression, and salt-and-pepper noise. Combining SVM with double-layer security makes the suggested model more resilient to various image processing threats. The brain image’s NROI section contains incorporated watermark information. Depending on the kind of medical imaging, the NROI component differs from one image to the next. Since each image’s NROI segment and embedding placements are unique, it is exceedingly challenging for an attacker to obtain important medical data that is concealed from view. The results of this experiment demonstrate the enhanced robustness and high imperceptibility of the suggested stenographic method, where 150 brain images were used. The PSNR value in this case is 67.81 dB, which is higher than the allowable amount, and an SSIM result on average equals 0.9999. The suggested strategy has several potential uses in telemedicine, as confirmed by the embedding method’s findings. However, because of the SVM model creation, the suggested model has a high initial computation complexity, which must be fixed. MR scans have been employed in the current investigation. In the future, the suggested model is planned to be investigated using other biomedical imaging modalities, including positron emission tomography (PET), computed tomography (CT), and ultrasound (US). Additionally, is proposed that NROIs be produced from medical images using deep learning (DL) models like convolutional neural networks (CNNs) rather than SVM to improve the classification accuracy of watermark embedding regions. Furthermore, hybrid watermarking techniques that combine LWT with additional transforms, such as discrete wavelet transform (DWT) or discrete cosine transform (DCT), may increase resilience against a range of attacks. By using a blockchain-based verification system, medical picture authentication can become even more secure and traceable, making it more resilient to advanced threats such adversarial attacks that target watermark recognition and deepfake-based manipulations. Lastly, future research may benefit from focusing on improving computational efficiency for real-time medical imaging applications.

## Data Availability

All data generated or analyzed during this study will be available from the corresponding author upon reasonable request.
